# Generation of a primary culture of chick embryo enterocytes to evaluate the effects of fumonisin B1 and deoxynivalenol on cell morphology, actin filaments and nuclei

**DOI:** 10.1371/journal.pone.0334395

**Published:** 2025-12-11

**Authors:** Jacqueline Uribe-Rivera, Maria de Jesús Nava-Ramirez, Saúl Escobedo-Aguirre, Juan Carlos Del Río-García, Samantha Jardón-Xicoténcatl, Carlos Gerardo García-Tovar, Ernesto Ávila-González, Gabriela Gomez-Verduzco

**Affiliations:** 1 Laboratorio 14 Alimentos, Micotoxinas y Micotoxicosis, Unidad de Investigación Multidisciplinaria, FES-Cuautitlán, Universidad Nacional Autónoma de México, Cuautitlán Izcalli, México; 2 Laboratorio 4 Morfología Veterinaria y Biología Celular, Unidad de Investigación Multidisciplinaria, FES-Cuautitlán, Universidad Nacional Autónoma de México, Cuautitlán Izcalli, México; 3 CEIEPAv (FMVZ-UNAM), Ciudad de México, México; 4 Departamento de Medicina y Zootecnia de Aves, Facultad de Medicina Veterinaria y Zootecnia, Universidad Nacional Autónoma de México, Ciudad Universitaria, Ciudad de México, México; Sindh Agriculture University, PAKISTAN

## Abstract

Fumonisin B1 (FB1) and deoxynivalenol (DON) are among the most impactful mycotoxins affecting poultry production. The negative effects of these mycotoxins on intestinal epithelial morphology and integrity have been previously described. However, cell culture methods using cells from species with different genetic backgrounds have been used to obtain these results. The objective of this work was to evaluate the damage caused by fumonisin B1 (FB1) and deoxynivalenol (DON) on cell morphology, actin filaments and nuclear morphology in primary enterocytes obtained from chick embryos via fluorescence microscopy. The results revealed that FB1 and DON did not induce actin filament depolymerization, and stress fibers remained intact under the concentrations of mycotoxins evaluated. However, the cytoarchitecture was disrupted in treated cells, leading to cell thinning and reduced viability, and this change affected one of the main characteristics of enterocytes, which is their ability to absorb nutrients. Damage to the integrity of the nucleus was also observed until its fragmentation, especially in cells treated with deoxynivalenol (DON). These results provide new insights into the mechanisms of action by which DON and FB1 exert their negative effects on chicken enterocytes, for which there was no previous information.

## Introduction

The gastrointestinal tract is involved in the absorption of nutrients from and the transportation and digestion of ingested food products. Moreover, it is home to a group of microorganisms, secretes endogenous substances, and, finally, protects against infectious and noninfectious agents [1,2]. Enterocytes are highly dynamic cells whose stable structure is required to maintain a state of homeostasis; the structural stability of enterocytes largely depends on their cytoskeleton. The cytoskeleton is composed of an organized and dynamic framework composed of actin filaments, intermediate filaments and microtubules, which are formed from the polymerization of proteins with less complex structures. The cytoskeleton, composed of actin filaments, intermediate filaments, and microtubules, is essential for maintaining enterocyte structure and function. Actin filaments, in particular, are responsible for generating contractile forces, supporting cellular architecture, and enabling mechanosensing. These filaments are anchored to junctional complexes, contributing to epithelial cohesion and selective permeability [3–5]. In addition, they act as the main component that promotes the generation of tractile and contractile forces in cells. The forces are generated by the polymerization of actin filaments (Arp2/3 complex) and by the sliding of myosin II along the actin filaments, respectively [6]. Therefore, studying the movement, structure and dynamics of filaments found in the cytoskeleton can provide insights into the functionality and viability of certain types of cells.

The intestinal epithelium can be compromised by the presence of different pathogens, which can be reach the intestine via the consumption of contaminated food [7–9]. One of the main consequences of fungal contamination is the production of mycotoxins. Mycotoxins are potentially toxic secondary metabolites produced by filamentous fungi that belong mainly to the genera *Aspergillus, Penicillium and Fusarium* [10]. Approximately 50–70% of animal feed is contaminated with mycotoxins [11]; specifically, this percentage in Central America is approximately 78%, of which between 68% and 78% of feed is contaminated with fusariotoxins [12]. Fusariotoxins are currently the most prevalent group of mycotoxins worldwide. Within this group, the mycotoxins that have increased in prevalence in recent years are fumonisins and deoxynivalenol [13]. This raises various questions about the mechanisms of action of these mycotoxins and the control methods applied in this area, especially in the context of poultry farming, for which many unknowns remain. The concentrations selected for FB1 (5–20 µM) and DON (0.1–1 µM) were chosen based on existing toxicological literature and documented contamination levels in poultry feed. According to guidance from the U.S. Food and Drug Administration (FDA), the maximum tolerated concentrations for poultry are 50 mg/kg for the combined total of FB1 and FB2, and 5 mg/kg for DON in finished feed [14]. Recent global mycotoxin surveys, including BIOMIN’s 2023 report, have recorded average fumonisin levels of 4.2 mg/kg (with peaks up to 83 mg/kg) and DON levels averaging 1.4 mg/kg in poultry diets [15]. Furthermore, controlled exposure studies in broilers have shown that subclinical doses of 10 mg/kg DON and 12 mg/kg FB1 can negatively impact gut morphology and intestinal microbiota composition [16,17]. Therefore, the in vitro doses employed in this study reflect both realistic field exposure ranges and standardized challenge levels commonly used in mechanistic toxicology assays.

Previous experimental studies have revealed that the toxins produced by *Fusarium* sp. affect mainly intestinal integrity; however, many of the mechanisms involved are still unknown, especially in birds. Thus, the generation of updated information about mycotoxins in intestinal cells is of vital importance. In this case, the use of primary cells allows better representation of the tissue of origin. Therefore, primary cells are ideal models for studying the cellular dynamics of a specific tissue. Until now, models established with mammalian intestinal epithelial cells have been commonly used, but these are often based on immortalized or cancer-derived lines; besides immortalized cell lines, which are mostly of carcinogenic origin, have been used in most *in vitro* studies; the replication cycle of immortalized cell lines is unlimited and the cell cycle is therefore altered, which can alter the results obtained from experiments using these cells [18,19]. In addition, it should be considered that the intestinal epithelial cell isolation protocols cannot be extrapolated from one animal to another, so research on primary intestinal cell isolation in birds itself is essential since primary cell isolation protocols have been established in mice and pigs; however, the protocols can vary [20].

Accordingly, the present study aimed to evaluate the effects of fumonisin (FB1) and deoxynivalenol (DON) on the morphology of chicken enterocytes through a novel and species-specific in vitro model, based on the isolation and primary culture of enterocytes from chicken embryos. This model was specifically developed to overcome the limitations of non-avian cell lines and to provide a biologically relevant platform for studying mycotoxin-induced intestinal damage in poultry. The study was conducted under specific pathogen-free conditions, with a particular focus on actin filament architecture and cellular viability. This innovative primary culture system represents a significant advancement for mechanistic research on mycotoxins in avian species.

## Materials and methods

### Primary culture of enterocytes isolated from chicken embryo

Ten embryonated chicken eggs *(Gallus gallus domesticus)* that had been incubated for 11 days and were free of specific pathogens were used; these eggs were donated by Diagnosticos Clínicos Veterinario SA de CV. The viability of the embryos was determined via the ovoscopy technique [21].

### Primary culture

According to legislation and protocols reported by CICUA (Comité Interno para el Cuidado y Uso de los Animales de la FMVZ de la UNAM); chick embryos were sacrificed by decapitation. The eggs were disinfected with sterile gauze soaked with a solution of Red Antibenzil Concentrate (1% benzalkonium chloride and 0.5% sodium nitrite) [22], and gently rubbing the obtuse pole of the egg, to have access to the air chamber. Then, the eggshell was gently cracked and the embryo was extracted with dissecting forceps.

An incision was made around the air chamber to visualize the embryo’s position. Using forceps, the embryo was grasped at the base of the head, carefully removed from the egg, and the yolk sac was separated. The dotted line indicates the orientation of the incision on the egg (blunt end) to proceed with the extraction.

Once extracted, the embryos were decapitated to extract the portion corresponding to the small intestine, and once extracted, they were collected in Petri dishes in sterile PBS at room temperature, inside which they were first cleaned to remove mesenteric fat and blood. The washes were performed twice, first with sterile PBS solution and then with basal rinsing medium (Medium 199 w/ Earle’s Mod. Salts w/ L-Glutamine w/ 1.25 g/l Sodium Bicarbonate (Cat. L0355- Biowest); Sterile Fetal Bovine Serum, free of mycoplasma (Cat. S1820- Biowest) added to 20%; penicillin- streptomycin 100x (L0022- Biowest); Gentamicin– 100 (Q-0040–013- Wittney); gentamicin–100 (Q-0040–013- Wittney).

The intestinal tissue was homogenized using 22 mm stainless steel mesh filters with a pore size of 120 μm to obtain the intestinal mucosa, which was corroborated through the use of several histological staining techniques such as Masson’s trichrome, silver, and hematoxylin–eosin staining [23]. The enzymatic homogenization of the collected tissue was subsequently carried out by adding 1.5 mL of trypsin/0.5% ethylenediaminetetraacetic acid (EDTA) for 10–15 min in Petri dishes of 40 × 12 mm in an incubator at 37°C in an atmosphere of 5% CO2 and 95% humidity; later, the enzymatic reaction was stopped by adding 2 mL of culture medium 199, which was supplemented with 10% FBS and antibiotics (penicillin (5000 IU) – streptomycin (5 μg/ml)) (maintenance medium) to incubate at 37°C in an atmosphere of 5% CO2 and 95% humidity for approximately 3–5 days to allow for cell proliferation and monolayer formation until it reached a confluence of between 90 and 100%.

### Cell morphology

Once the monolayer was established and reached 100% confluence, a cell passage was performed. The disaggregated cells were then seeded at a concentration of 3 × 10⁵ cells per well in a 24-well culture plate and incubated at 37°C in a humidified atmosphere containing 5% CO₂ and 95% relative humidity until 90% confluence was achieved. Subsequently, the plate was exposed to various concentrations of FB1 and DON. The appropriate volumes of FB1 and DON stock solutions were added to each experimental well, while control wells received an equivalent volume of sterile distilled water, corresponding to the highest volume used in the treatment groups. Morphological assessments were performed at 24 and 48 hours using an inverted microscope (UNICO®).

### Evaluation of the actin cytoskeleton

For the evaluation of the actin cytoskeleton, the direct fluorescence technique was performed 48 hours after cell seeding. Cells were cultured on 12 mm circular glass coverslips and subjected to FB1, DON, or vehicle (control) exposure. Following treatment, cells were fixed with 10% neutral buffered formalin in PBS for 20 minutes, washed three times with PBS, and permeabilized with 0.5% Triton X-100 for 5 minutes. For the detection of actin filaments, phalloidin conjugated with tetramethylrhodamine isothiocyanate (Phalloidin-TRITC labeled, Sigma) (diluted 1:100 in PBS) was added to the cells and incubated for 20 min in an incubator with high humidity in the dark, followed by 2 washes with PBS. Finally, the samples were washed with deionized water and mounted on slides using special 4’6-diamino-2-phenylindole (DAPI) mounting medium for fluorescence (Ultracruzᵀᴹ Mounting Medium for Fluorescence, Santa Cruz). The samples were observed and analyzed with a Carl Zeiss brand fluorescence microscope, model Axioskop 40, coupled to a color camera (Evolution VF). The images were analyzed using the ImageJ® software.

### Toxin solutions

Fumonisin B1 (BIOPURE) and deoxynivalenol (Merck) were diluted in sterile distilled water to prepare stock solutions with initial concentrations of 4156.1 μM and 674.94 μM, respectively. From these, aliquots were used to generate three exposure levels—low (LD), medium (MD), and high (HD)—for each mycotoxin. The selected concentration ranges reflect environmentally relevant levels of contamination reported in poultry feed, particularly in tropical regions, and were based on previous toxicological studies evaluating intestinal cytotoxicity in poultry and swine models [24–27]. These levels were chosen to model sublethal to cytotoxic effects in vitro. Exposure times of 24 and 48 hours were applied to simulate acute and subacute effects on cell morphology and viability. These exposure conditions were designed to mimic short-term high-risk contamination events rather than chronic low-dose intake.

#### Experimental desing.

An experimental design was established with three treatment groups: fumonisin B1 (FB1), deoxynivalenol (DON), and a control group (C). For both FB1 and DON groups, three concentrations were tested, each with three replicate wells, including the control group. Treatments were applied in 24-well cell culture plates (Costar®), and the entire experimental setup was independently replicated 6 times. Table 1 shows the toxin concentration levels for each treatment with the suggested concentration in parts per million (ppm), as well as its equivalent micromolar concentration. Similarly, the number of replicates treated with each concentration is shown, and it is important to mention that the values in the table represent the experimental layout used per plate. The concentrations were selected to simulate acute (24 h) and subacute (48 h) exposure scenarios relevant to typical contamination levels found in poultry feed [24–27].

**Table 1:** Distribution scheme of the concentration of each mycotoxin with respect to the suggested experimental design in 24-well culture plates.

Treatments	ppm	µM	No. of replicates
**Fumonisin B1- low dose (FB1-LD)**	1	1.4	3
**Fumonisin B1- medium dose (FB1-MD)**	3	4.2	3
**Fumonisin B1- high dose (FB1-HD)**	5	6.9	3
**Deoxynivalenol- low dose (DON- LD)**	0.5	1.7	3
**Deoxynivalenol- medium dose (DON-MD)**	1	3.4	3
**Deoxynivalenol- high dose (DON- HD)**	3	10.1	3
**Control**	No addition of mycotoxin	No addition of mycotoxin	6

#### Statistical analysis.

Given the nonparametric distribution of the data, medians and their standard errors were calculated using the bootstrap resampling method (1,000 iterations). The standard error of the median, obtained via bootstrap, is referred to as SE-mboot. Group comparisons were subsequently performed using the Mann–Whitney U test, with a significance level set at p < 0.05. All statistical analyses were conducted using Minitab® software (version 16), and confidence intervals were set at 95%.

#### Ethical considerations.

According to NOM-062-ZOO-1999, AVMA guidelines and animal welfare, chick embryos were sacrificed by decapitation. As chick embryos older than 14 d can experience pain, decapitation was recommended as a humane method of euthanasia, avian embryos are not considered as and international guidelines, chick embryos ≤17 days of incubation do not require ethics committee approval. All procedures followed ethical principles and good scientific practice.

## Results and discussion

### Primary cell culture

A protocol was developed for the isolation and maintenance of enterocytes derived from chicken embryos with the aim of establishing a novel model to study the effects and biochemical mechanisms involved in avian mycotoxicosis. This condition typically begins with the ingestion of contaminated feed, which initially interacts with the intestinal epithelium. The primary objective was to generate a primary culture using a simplified procedure that would facilitate the removal of undesired tissue. This was achieved by isolating intestines during the early days of incubation. In previously reported studies, intestinal tissue was typically collected after hatching or during the final days of incubation, close to hatching, which presents greater technical challenges for dissection and increases the risk of bacterial contamination during culture processing [28–32].

**Fig 1:**
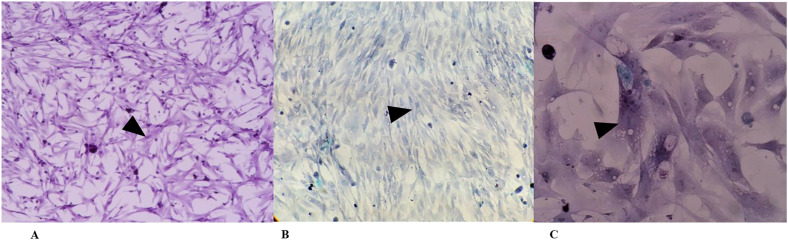
Morphological evaluation of primary enterocyte cultures from chicken embryos using different histological stains. Cell transition from polygonal to spindle-shaped morphology with elongated cell borders. A) Hematoxylin and eosin staining; B) Modified silver staining; C) Masson’s trichrome staining. Panels A and B: 40x magnification; Panel C: 100 × magnification.

To determine the type of cell that was isolated using the primary culture protocol, 3 histological stains were performed (Fig 1). According to the hematoxylin‒eosin staining results (Fig 1A), the morphology observed in the cells isolated from the intestine of 11-day-old chick embryos corresponded to that of enterocytes and coincided with what was previously reported by other authors using other methodologies [23]. These studies indicate that, between days 3 and 11 of maintenance of the primary culture, depending on the protocol applied, the cell morphology changed from a rounded shape to a polygonal or spindle-shaped morphology with extensions. Notably, most enterocyte cultures have been established with cells from one-day-old chicks, typical enterocyte morphology is not observed until 11 days of culture [23]. However, in this case, between days 5 and 7 after the primary culture, the morphology of enterocytes reported, and therefore the staining results were corroborated.

In the case of modified silver staining (Fig 1B) and Masson’s trichrome staining (Fig 1C), the isolated cells did not correspond to reticular fibers associated with connective tissue, which were black with a yellowish background due to impregnation with silver particles [33], whereas in the trichrome staining, collagen or elastic fibers were detected with red or green staining [34], which was not observed in the cultures; this is why, according to these findings, the cells formed in the primary culture corresponded to epithelial cells.

### Cell morphology

As shown in Table 2, spindle-shaped morphology significantly decreased following FB1 exposure, with adjusted p-values of 0.0134 and 0.0087, particularly at 48 hours post-challenge compared to 24 hours. This reduction was most notable in the medium-dose group (FB1-MD), where spindle morphology decreased by up to 1.75-fold at 48 hours. Another key observation was a cytomorphological alteration characterized by cellular thinning, which increased in the low- (FB1-LD) and high-dose (FB1-HD) groups, with adjusted p-values of 0.0183 and 0.0032, showing increases of up to 1.75- and 2-fold, respectively, at 48 hours versus 24 hours.

**Table 2:** Evaluation of cell morphology after challenge with FB1 at 24 and 48 hours.

Time	24 h				48 h			
Concentration	Control	Fumonisin B1- low dose (FB1-LD)	Fumonisin B1- medium dose (FB1-MD)	Fumonisin B1- high dose (FB1-HD)	Control	Fumonisin B1- low dose (FB1-LD)	Fumonisin B1- medium dose (FB1-MD)	Fumonisin B1- high dose (FB1-HD)
Spindle-shaped morphology	4 ± 0.33^a^	4 ± 0.34^a^	3.5 ± 0.42^a^	3 ± 0.34^a^	3.5 ± 0.41^a^	3 ± 0.33^a^	2 ± 0.34^b^	1 ± 0.34^b^
Letal cytomorphological change	0 ± 0.15^a^	1 ± 0.33^b^	2 ± 0.42^a^	2 ± 0.13^b^	0 ± 0.34^a^	2 ± 0.32^a^	2 ± 0.56^a^	3.5 ± 0.42^a^
Cellular debris	0 ± 0.33^a^	1 ± 0.15^a^	2 ± 0.34^a^	3 ± 0.35^b^	0.5 ± 0.41^a^	1 ± 0.33^a^	2.5 ± 0.42^a^	4 ± 0.14^a^
Loss of cell confluence	1 ± 0.33^a^	0.5 ± 0.42^b^	2 ± 0.33^a^	2 ± 0.34^b^	1 ± 0.14^a^	2 ± 0.42^a^	2 ± 0.32^a^	4 ± 0.32^a^

SEm (boot). The different letters indicate significant differences between the same treatment at different times (p < 0.05) according to the Mann–Whitney U test with correction for ties.

Monolayer integrity assessment also revealed a marked loss of cell confluence in the FB1-LD and FB1-HD groups (adjusted p = 0.0134 and 0.0087), with reductions of up to twofold at 48 hours, more pronounced than at 24 hours. Additionally, an increase in cellular debris was observed, particularly in the FB1-HD group (adjusted p = 0.0073).

Table 3 shows the effects of DON on the same metrics evaluated above. The morphology (p-adjusted = 0.0498) revealed that the spindle size decreased by 1.75 times in the DON-HD group compared to the DON-MD group at 48 h than at 24 h. In this case, the thinning of the cells was more pronounced with respect to the FB1 treatment, with p-adjusted values of 0.0383 and 0.0248. Additionally, this effect was more pronounced for the DON-LD and DON-HD groups (decrease of up to 4 times in the 48 h reading). Compared with that of the control group, the cellular confluence, with p-adjusted values of 0.0244 and 0.0239, decreased to 4 times in the DON-HD group, which was related to the increase in the amount of cellular debris at the same dose (p-adjusted values = 0.0439 and 0.0134, respectively).

**Table 3:** Evaluation of cell morphology after DON challenge at 24 and 48 hours.

Time	24 h				48 h			
Concentration	Control	Deoxynivalenol – low dose (FB1-LD)	Deoxynivalenol – medium dose (FB1-MD)	Deoxynivalenol – high dose (FB1-HD)	Control	Deoxynivalenol – low dose (FB1-LD)	Deoxynivalenol – medium dose (FB1-MD)	Deoxynivalenol – high dose (FB1-HD)
Spindle-shaped morphology	4 ± 0.15^a^	4 ± 0.34^a^	3.5 ± 0.48^a^	2 ± 0.16^a^	4 ± 0.14^a^	3.5 ± 0.42^a^	2 ± 0.35^b^	2 ± 0.37^b^
Letal cytomorphological change	0 ± 0.35^a^	0.5 ± 0.46^b^	2.5 ± 0.47^a^	4 ± 0.15^b^	0.5 ± 0.41^a^	2 ± 0.55^a^	3.5 ± 0.42^a^	4 ± 0.32^a^
Cellular debris	0 ± 0.32^a^	1 ± 0.39^a^	2.5 ± 0.41^a^	4 ± 0.34^b^	0.5 ± 0.41^a^	2.5 ± 0.69^a^	4 ± 0.33^a^	4 ± 0.14^a^
Loss of cell confluence	0 ± 0.33^a^	1 ± 0.37^b^	2.5 ± 0.41^a^	3.5 ± 0.42^b^	0.5 ± 0.42^a^	2 ± 0.39^a^	3.5 ± 0.41^a^	4 ± 0.33^a^

SEm (boot). The different letters indicate significant differences between the same treatment at different times (p < 0.05) according to the Mann–Whitney U test with correction for ties.

**Fig 2:**
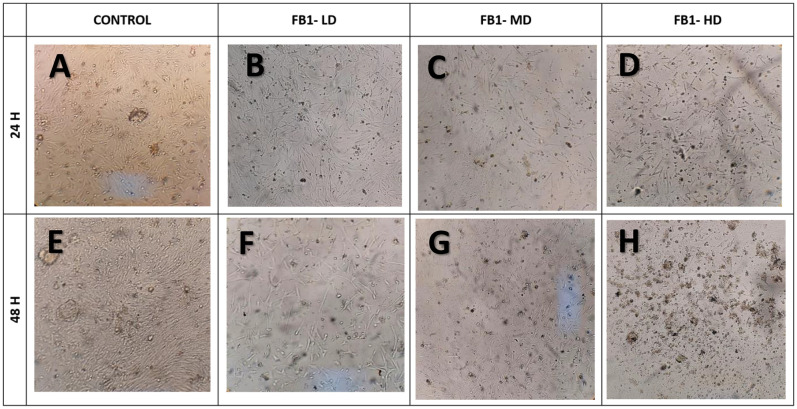
Evaluation of cell morphology in primary enterocyte cultures from chicken embryos exposed to different concentrations of FB1. Three FB1 concentrations are shown at 24 hours (B–D) and 48 hours (F–H). Control no mycotoxin added; FB1-LD 1.4 µM (low); FB1-MD 4.2 µM (medium); FB1-HD 6.9 µM (high) concentrations. Images obtained using an inverted microscope.

**Fig 3:**
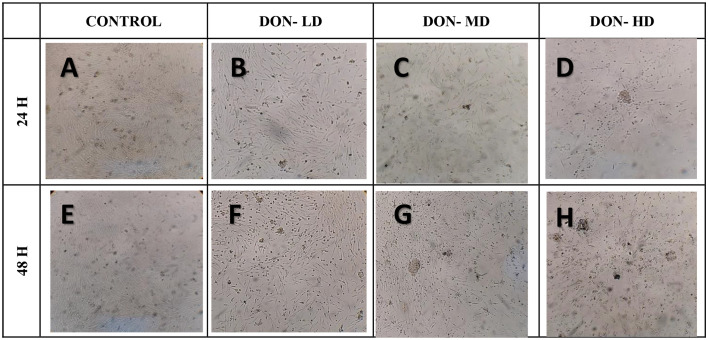
Evaluation of cell morphology in primary enterocyte cultures from chicken embryos exposed to different concentrations of DON. Three DON concentrations are shown at 24 hours (B–D) and 48 hours (F–H). Control no mycotoxin added; DON-LD – 1.7 µM (low); DON-MD 3.4 µM (medium); DON-HD 10.1 µM (high) concentrations. Images obtained using an inverted microscope.

In the case of the FB1 and DON treatments, the damage to the monolayer was directly proportional to the concentration and exposure time of the mycotoxins. Cells exposed to these toxins initially maintained a spindle-shaped morphology at 24 and 48 hr post-challenge (Tables 2 and 3); however, morphological alterations became more evident as toxin concentrations increased, particularly in the DON-treated group. Cells exposed to DON exhibited structural thinning as early as 24 hours in the DON-LD group (Fig 3). Similar alterations were observed with FB1 exposure, notably in the FB1-MD group (Fig 2). Monolayer damage was more severe with DON, with complete monolayer destruction and abundant cellular debris observed at 48 hours (Fig 3). The comparative results suggest that, under the conditions evaluated in this study, DON induces greater cytotoxic damage than FB1.

Three DON concentrations are shown at 24 hours (B–D) and 48 hours (F–H).

Control – no mycotoxin added; DON-LD – 1.7 µM; DON-MD – 3.4 µM; DON-HD – 10.1 µM. Cellular debris is indicated by black arrows. Structural thinning is indicated by black arrowheads. Images obtained using an inverted microscope. A decrease in cell proliferation induced by FB1 has been reported in human umbilical vein endothelial cells and is associated with its effects on cytoarchitecture [35]. Therefore, this mechanism could be related to the decrease in the size of the enterocytes, which gradually decreases until the cells become cellular debris, which in turn is related to the viability of the cells treated as part of a lethal cytomorphological change [36].

### Evaluation of the actin cytoskeleton

**Fig 4:**
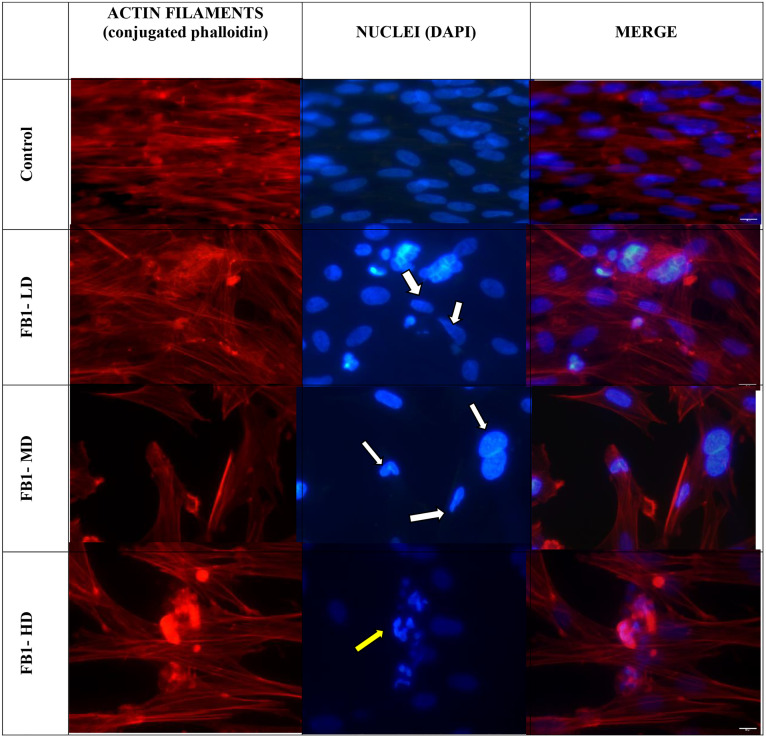
Evaluation of actin cytoskeleton and nuclear integrity following exposure to fumonisin B1. Three FB1 concentrations are shown at 48 hours. Control – no mycotoxin added; FB1-LD 1.4 µM (low); FB1-MD 4.2 µM (medium); FB1-HD 6.9 µM (high) concentrations. Nuclear damage ranged from alterations in nuclear shape and diameter (white arrows) to advanced fragmentation (yellow arrow). Fluorescence microscopy, 40 × magnification.

Statistical analysis of the fluorescence data from cells treated with FB1 revealed that there was a significant difference between the three FBI-treated groups and the control group, which could be correlated with a cumulative effect of the mycotoxin on the loss of typical morphology; the size of enterocytes decreased by up to 3 times (p-adjusted = 0.0035 in both cases). In turn, the comparison between the treatment groups and the control group, especially the FB1-HD treatment groups, indicated that FB1 treatment induced greater cellular morphological damage, which was related to the thinning of the cells (p-adjusted = 0.0063). The progressive decrease in cell confluence followed the same trend (p-adjusted = 0.0092 and 0.0228), which supports the observation that the adverse effect of FB1 on cell cohesion was up to 3 times greater than that of the control treatment. Regarding nuclear integrity (low dose: p-adjusted = 0.0073; medium dose: 0.0067; high dose: 0.0045), significant progressive differences were observed between the different doses applied and the control treatment, highlighting a dose-dependent effect of the treatment. No changes were observed in actin filament polymerization across FB1-treated groups, suggesting that cytoskeletal integrity was preserved (all p values adjusted > 0.28) (Table 4) (Fig 4).

**Table 4:** Evaluation of changes in actin filaments and nuclei after challenge with FB1 at 48.

Time	48 h			
Concentration	Control	Fumonisin B1- low dose (FB1-LD)	Fumonisin B1- medium dose (FB1-MD)	Fumonisin B1- high dose (FB1-HD)
Spindle- shaped characteristic	4 ± 0.34^a^	3 ± 0.33^b^	2 ± 0.34^c^	1 ± 0.33^d^
Lethal cytomorphological change	0 ± 0.14^c^	1.5 ± 0.42^b^	2 ± 0.35^b^	3.5 ± 0.42^a^
Loss of cell confluence	1 ± 0.13^c^	2 ± 0.34^b^	2 ± 0.21^b^	3.5 ± 0.47^a^
Depolymerization of actin filaments	0 ± 0.16^a^	0 ± 0.33^a^	0 ± 0.32^a^	0.5 ± 0.42^a^
Loss of nuclear integrity	0 ± 0.15^c^	1 ± 0.33^b^	1.5 ± 0.65^ab^	3 ± 0.61^a^

Median ± SE Different letters in the same row indicate significant differences (p < 0.05) according to the Mann–Whitney U test with correction for ties.

In the evaluation of the effect of DON on cell integrity, a loss of the characteristic cell shape was observed in the DON-MD and DON-HD groups compared to the control group, indicating a progressive effect of the mycotoxin treatment (p adjusted = 0.0029 in both cases). In the same way, in relation to the lethal cytomorphological change, the comparison between all the doses and the control treatment was statistically significant, with a progressive difference in the treatments associated with the progression of cellular damage (p-adjusted ≤ 0.0017). In the case of cell confluence, a statistically significant decrease was observed between the different doses and the control treatment, especially between the control treatment and DON-HD (control against DON-LD (p-adjusted = 0.0035), control against DON-MD (0.0035), and control against DON-HD (0.003)). The integrity of the nuclear structure also significantly differed with respect to the control treatment (control against DON-LD (p-adjusted = 0.0037), control against DON-MD (0.0035), and control against DON-HD (0.003)). Similarly, DON exposure did not result in significant disruption of filament polymerization or organization, regardless of concentration (all p values adjusted greater than 0.64) (Table 5).

**Table 5:** Evaluation of changes in actin filaments and nuclei after DON challenge at 48 h.

Time	48 h			
Concentration	Control	Deoxynivalenol- low dose (DON- LD)	Deoxynivalenol- medium dose (DON- MD)	Deoxynivalenol- high dose (DON- HD)
Spindle- shaped characteristic	4 ± 0.34^a^	3.5 ± 0.44^ab^	2 ± 0.33^c^	2 ± 0.33^c^
Lethal cytomorphological change	0 ± 0.34^d^	2 ± 0.33^c^	3 ± 0.19^b^	4 ± 0.15^a^
Loss of cell confluence	0 ± 0.33^d^	2 ± 0.33^c^	3 ± 0.32^ab^	4 ± 0.16^a^
Depolymerization of actin filaments	0 ± 0.34^a^	0.5 ± 0.41^a^	0 ± 0.33^a^	0 ± 0.34^a^
Loss of nuclear integrity	0 ± 0.33^d^	2.5 ± 0.41^c^	3 ± 0.34^ab^	4 ± 0.13^a^

Median ± SE Different letters in the same row indicate significant differences (p < 0.05) according to the Mann–Whitney U test with correction for ties.

**Fig 5:**
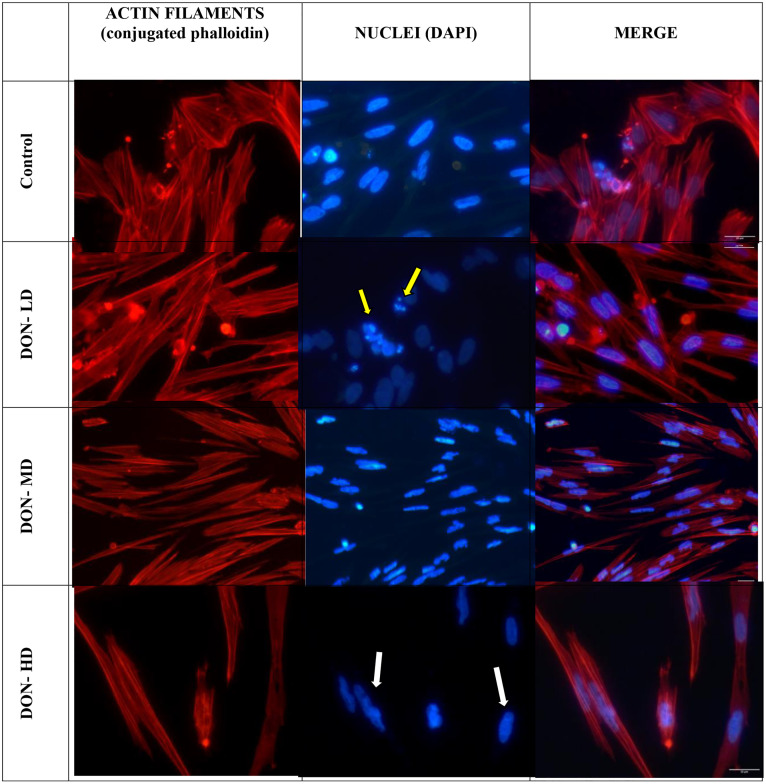
Evaluation of actin cytoskeleton and nuclear integrity following exposure to deoxynivalenol. Three DON concentrations are shown at 48 hours. Control no mycotoxin added; DON-LD 1.7 µM (low); DON-MD 3.4 µM (medium); DON-HD 10.1 µM (high) concentrations. Nuclear damage ranged from alterations in nuclear shape and diameter (white arrows) to advanced nuclear fragmentation (yellow arrow). Fluorescence microscopy, 40 × magnification.

The fluorescence microscopy results revealed that the integrity of stress fibers was maintained in all DON-treated groups (Fig 5), an effect not observed in groups treated with FB1, which induces cytomorphological changes in primary cultures of human hepatocytes. Cellular thinning observed in our study could be associated with cytoskeletal rearrangements and focal cytoplasmic retraction. Shen *et al*. (2019) [37] reported that AFB1 exposure in IPEC-J2 cells induces significant cytomorphological changes, including elongation and a thinner appearance of cells, alongside the disruption of actin filaments. Although AFB1 was not part of our experimental design, these findings support the idea that DON and FB1 may similarly alter cytoskeletal dynamics, resulting in comparable morphological effects [38].

However, nuclear staining revealed damage to the integrity of the organelle, which results in a loss of structural continuity and nuclear positioning, progressing to chromatin condensation until fragmentation, ending with cell death (Fig 5, yellow arrows).

FB1 acts by altering the synthesis of sphingolipids through the inhibition of the enzyme ceramide synthase, which leads to the accumulation of sphingoid bases such as sphinganine and sphingosine [26,39,40]. This accumulation has been related mainly to the increase in the production of free radicals, which leads to lipid peroxidation and an increase in the expression of proteins involved apoptotic processes, such as Caspase-3 and tumor necrosis factor (TNF) [41–43], as well as the activation of nuclear receptors, such as NXR, AHR, CAR and PXR [44,45]. In human esophageal carcinoma cells, FB1 causes a decrease in cell viability, externalization of phosphatidylserine, an increase in the expression of BAX proteins (which regulate the mitochondrial pathway of apoptosis in epithelial cells), and the induction of DNA fragmentation [46]. Unfortunately, studies aimed at understanding the mechanism of action of fumonisins have been performed mostly in mice and cell lines from human carcinogenic tissues.

In the case of deoxynivalenol, (Fig 5) fluorescence microscopy revealed that although the actin filaments and stress fibers did not change in response to DON treatment, the elongation of the nuclei was mainly observed after DON-LD treatment, which intensified in the DON-HD treatment group, increasing in severity with increasing dose. Compared with FB1 treatment, DON treatment resulted in much greater thinning of the cells, in addition to a clear effect on the integrity of the nuclei, as evidenced by the loss of normal morphology. DON exerts its effects by altering protein synthesis when it binds to the 60s ribosomal fraction, preventing it from binding the 40 s fraction, which has repercussions directly on the translation of the messenger RNA. This consequently generates alterations in the elongation and termination stages of protein synthesis, as well as alterations in the ribosomal conformation [47–49]. In turn, the effect of DON has also been related to a decrease in cell viability through an increase in oxidative stress due to increases in the levels of 8-HdG and reactive oxygen species (ROS) in addition to the formation of nitrotyrosine [50–52]. These processes have also been described in the context of carcinogenesis induced by mutations in DNA and abnormalities at the chromosomal level. In addition, as with fumonisins, the generation of apoptotic mechanisms related to DON has also been described, mainly by phosphorylation of MAPK (mitogen-activated protein kinase) and by inhibition of the first cleavage in the zygote under challenge with high concentrations of DON, while at low levels, oxidative damage and DNA damage have been observed to affect early embryonic development as well as maturation [53–56].

The investigation of mycotoxins using techniques such as cell culture is essential for understanding the origin of their toxic effects in living organisms, particularly in poultry [57]. This method offers significant advantages, including the ability to evaluate the mechanisms of action and toxicity of mycotoxins in a controlled environment. This allows for the identification of potential mitigation strategies without compromising animal health, in accordance with current animal welfare regulations and cost-reduction practices [58]. Table 6 summarizes the most relevant findings through a comparative analysis of the effects of fumonisin B1 and deoxynivalenol. It was found that DON induces more pronounced nuclear damage compared to fumonisin B1, whose most notable effect was the loss of cell viability. No alterations in the actin cytoskeleton were observed in any treatment group.

**Table 6:** Summary of structural and morphological alterations observed in chicken embryo enterocytes following exposure to fumonisin B1 (FB1) and deoxynivalenol (DON).

Parameter	Control	DON	Fumonisin
Cell morphology	Preserved (fusiform)	Preserved fusiform shape	Preserved fusiform shape
Cytomorphological alterations	None	Severe alterations with prominent cell debris	Moderate alterations with increased cell debris
Cellular confluence	Maintained	Slightly reduced	Significantly reduced
Actin filament polymerization	Intact	Intact	Intact
Nuclear integrity	Preserved	Severe loss of nuclear integrity	Preserved

These results suggest that even low-level exposure to deoxynivalenol and fumonisin B1 can compromise intestinal integrity in poultry, potentially affecting nutrient absorption and overall productive performance in commercial flocks. Although the cytotoxic effects of mycotoxins are well documented in mammalian cells, data on avian enterocytes remain limited. This study provides novel insights into the differential responses of chicken intestinal cells to DON and FB1 exposure, highlighting the need to use species-specific models.

Further research is required to establish in vivo correlations and to explore the dynamics of tight junction proteins in order to better understand the functional implications for intestinal permeability. Our research group is currently conducting studies in this area. Recognizing the distinct cellular footprints that these mycotoxins leave on enterocytes may support the development of targeted and sustainable mitigation strategies in poultry nutrition.

The details of the statistical analysis of morphological differences after the addition of FB1 and DON are found in the Supporting Information attached as:

Supporting Information 1 (S1 File). Summary of the Mann – Whitney analysis carried out for the Evaluation of the Cell Morphology in the treatment to which FB1 was added.

Supporting Information 2 (S2 File). Summary of the Mann – Whitney analysis carried out for the Evaluation of the cell Morphology in the treatment to which DON was added.

Supporting Information 3 (S3 File). Summary of the Mann – Whitney analysis carried out for the Evaluation of the integrity of actin filaments and nuclei by immunofluorescence with the addition of FB1.

Supporting Information 4 (S4 File). Summary of the Mann–Whitney analysis for the evaluation of actin filament and nuclear integrity by immunofluorescence following DON exposure.

## Conclusion

This study contributes to the development of an in vitro model specifically designed to study the early cellular effects of mycotoxins in avian enterocytes. While the findings demonstrate that both fumonisin B1 (FB1) and deoxynivalenol (DON) can induce cytotoxic changes at low concentrations, the results should be interpreted within the limitations of an in vitro system. The observed nuclear damage caused by DON and the reduction in monolayer viability associated with FB1 highlight distinct modes of action that warrant further in vivo investigation. The partial conservation of cellular morphology at low concentrations suggests a dose- and time-dependent toxicity threshold. These findings underscore the importance of using species-specific models to better understand the toxicological impact of mycotoxins in poultry.

## Supporting information

S1 File Summary of the Mann – Whitney analysis carried out for the Evaluation of the Cell Morphology in the treatment to which FB1 was. (DOCX)

S2 File Summary of the Mann – Whitney analysis carried out for the Evaluation of the cell Morphology in the treatment to which DON was added. (DOCX)

S3 File Summary of the Mann – Whitney analysis carried out for the Evaluation of the integrity of actin filaments and nuclei by immunofluorescence with the addition of FB1. (DOCX)

S4 File Summary of the Mann–Whitney analysis for the evaluation of actin filament and nuclear integrity by immunofluorescence following DON exposure. (DOCX)
