# Supplementary material for: Generation of a primary culture of chick embryo enterocytes to evaluate the effects of fumonisin B1 and deoxynivalenol on cell morphology, actin filaments and nuclei
Source: PLoS One. 2025 Dec 11;20(12):e0334395. doi: 10.1371/journal.pone.0334395 (PMC12697969; doi:10.1371/journal.pone.0334395)
Supplement: S1 File — (DOCX) [file pone.0334395.s001.docx]

| **Variable evaluated** | **Comparison** | **N1/N2** | **Median 1** | **Median 2** | **ETA1–ETA2** | **95.5% CI** | **W** | **p-value** | | **Adjusted p-value** | |
| --- | --- | --- | --- | --- | --- | --- | --- | --- | --- | --- | --- |
| Spindle-shaped morphology | b vs b₁ | 6 | 4.0 | 3.0 | -0.0 | (-0.000; 1.000) | 45.0 | | 0.3785 | | 0.3109 |
| Spindle-shaped morphology | m vs m₁ | 6 | 3.5 | 2.0 | 1.0 | (0.000; 2.000) | 54.0 | | 0.0202 | | 0.0134 |
| Spindle-shaped morphology | a vs a₁ | 6 | 3.0 | 1.0 | 1.0 | (1.000; 2.000) | 55.0 | | 0.0131 | | 0.0087 |
| Spindle-shaped morphology | c vs c₁ | 6 | 4.0 | 3.5 | 0.0 | (-1.000; 1.000) | 42.0 | | 0.6889 | | 0.6404 |
| Lethal cytomorphological change | b vs b₁ | 6 | 1.0 | 2.0 | -1.0 | (-2.000; -0.000) | 25.0 | | 0.0306 | | 0.0183 |
| Lethal cytomorphological change | m vs m₁ | 6 | 2.0 | 2.0 | -0.5 | (-2.000; 1.001) | 34.0 | | 0.4712 | | 0.4372 |
| Lethal cytomorphological change | a vs a₁ | 6 | 2.0 | 3.5 | -2.0 | (-2.000; -1.000) | 21.0 | | 0.0051 | | 0.0032 |
| Lethal cytomorphological change | c vs c₁ | 6 | 0.0 | 0.0 | 0.0 | (-1.0001; 0.0004) | 36.0 | | 0.6889 | | 0.5948 |
| Cellular debris | b vs b₁ | 6 | 1.0 | 1.0 | -0.0 | (-1.0004; 0.0002) | 31.0 | | 0.2298 | | 0.1138 |
| Cellular debris | m vs m₁ | 6 | 2.0 | 2.5 | -1.0 | (-2.000; -0.000) | 27.0 | | 0.0656 | | 0.0379 |
| Cellular debris | a vs a₁ | 6 | 3.0 | 4.0 | -1.0 | (-2.0001; -0.9996) | 23.0 | | 0.0131 | | 0.0073 |
| Cellular debris | c vs c₁ | 6 | 0.0 | 0.5 | 0.0 | (-1.000; 1.000) | 36.0 | | 0.6889 | | 0.6404 |
| Loss of cell confluence | b vs b₁ | 6 | 0.5 | 2.0 | -1.0 | (-2.000; -0.000) | 24.0 | | 0.0202 | | 0.0134 |
| Loss of cell confluence | m vs m₁ | 6 | 2.0 | 2.0 | -1.0 | (-1.000; 0.000) | 29.0 | | 0.1282 | | 0.0689 |
| Loss of cell confluence | a vs a₁ | 6 | 2.0 | 4.0 | -1.0 | (-2.000; -1.000) | 23.0 | | 0.0131 | | 0.0087 |

Supporting Information 1 (S1). Summary of the Mann–Whitney analysis carried out for the Evaluation of the Cell Morphology in the treatment to which FB1 was added.

Comparison column: A24/A48- FB1-HD challenge at 24 or 48 h; M24/ M48- FB1-MD challenge at 24 or 48 h; B24/ B48-FB1-BD challenge at 24 or 48 hours;

C24/C48- control treatment without addition of mycotoxin; vs- versus or comparison.
