# Supplementary material for: Generation of a primary culture of chick embryo enterocytes to evaluate the effects of fumonisin B1 and deoxynivalenol on cell morphology, actin filaments and nuclei
Source: PLoS One. 2025 Dec 11;20(12):e0334395. doi: 10.1371/journal.pone.0334395 (PMC12697969; doi:10.1371/journal.pone.0334395)
Supplement: S2 File — (DOCX) [file pone.0334395.s002.docx]

Supporting Information 2 (S2). Summary of the Mann – Whitney analysis carried out for the Evaluation of the cell Morphology in the treatment to which DON was added.

| **Variable evaluated**  ANNEX 1. Summary of the Mann–Whitney analysis for the evaluation of cell morphology in the treatment with FB1 | **Comparison** | **N1/N2** | **Median 1** | **Median 2** | **ETA1–ETA2** | | **95.5% CI** | **W** | **p-value** | **Adjusted p-value** |
| --- | --- | --- | --- | --- | --- | --- | --- | --- | --- | --- |
| Spindle-shaped morphology | b vs b₁ | 6 | 4.0 | 3.5 | | 0.0 | (-1.000; 1.000) | 42.0 | 0.6889 | 0.6404 |
| Spindle-shaped morphology | m vs m₁ | 6 | 3.5 | 2.0 | | 1.0 | (0.000; 2.000) | 51.0 | 0.0656 | 0.0498 |
| Spindle-shaped morphology | a vs a₁ | 6 | 2.0 | 2.0 | | -0.0 | (-1.000; 1.000) | 38.5 | 1.0000 | 1.0000 |
| Spindle-shaped morphology | c vs c₁ | 6 | 4.0 | 4.0 | | -0.0 | (-0.0002; 0.0002) | 39.0 | 1.0000 | 1.0000 |
| Lethal cytomorphological change | b vs b₁ | 6 | 0.5 | 2.0 | | -1.0 | (-3.000; -0.000) | 26.0 | 0.0453 | 0.0383 |
| Lethal cytomorphological change | m vs m₁ | 6 | 2.5 | 3.5 | | -1.0 | (-2.000; -0.000) | 25.5 | 0.0374 | 0.0248 |
| Lethal cytomorphological change | a vs a₁ | 6 | 4.0 | 4.0 | | 0.0 | (-0.0004; 1.0001) | 42.0 | 0.6889 | 0.5948 |
| Lethal cytomorphological change | c vs c₁ | 6 | 0.0 | 0.5 | | 0.0 | (-1.000; 1.000) | 36.0 | 0.6889 | 0.6404 |
| Cellular debris | b vs b₁ | 6 | 1.0 | 2.5 | | -1.0 | (-3.000; -0.000) | 26.5 | 0.0547 | 0.0439 |
| Cellular debris | m vs m₁ | 6 | 2.5 | 4.0 | | -1.0 | (-2.000; -0.000) | 24.0 | 0.0202 | 0.0134 |
| Cellular debris | a vs a₁ | 6 | 4.0 | 4.0 | | 0.0 | (-1.0001; 0.0004) | 36.0 | 0.6889 | 0.5948 |
| Cellular debris | c vs c₁ | 6 | 0.0 | 0.5 | | 0.0 | (-1.000; 1.000) | 36.0 | 0.6889 | 0.6404 |
| Loss of cell confluence | b vs b₁ | 6 | 1.0 | 2.0 | | -1.0 | (-2.000; -0.000) | 25.0 | 0.0306 | 0.0244 |
| Loss of cell confluence | m vs m₁ | 6 | 2.5 | 3.5 | | -1.0 | (-2.000; 0.000) | 25.5 | 0.0374 | 0.0239 |
| Loss of cell confluence | a vs a₁ | 6 | 3.5 | 4.0 | | 0.0 | (-1.000; 1.000) | 36.0 | 0.6889 | 0.6404 |

Comparison column: A24/A48- DON-HD challenge at 24 or 48 h; M24/ M48-DON-MD challenge at 24 or 48 h; B24/ B48-DON-BD challenge at 24 or 48 hours;

C24/C48- control treatment without addition of mycotoxin; vs- versus or comparison.
