# Supplementary material for: Generation of a primary culture of chick embryo enterocytes to evaluate the effects of fumonisin B1 and deoxynivalenol on cell morphology, actin filaments and nuclei
Source: PLoS One. 2025 Dec 11;20(12):e0334395. doi: 10.1371/journal.pone.0334395 (PMC12697969; doi:10.1371/journal.pone.0334395)
Supplement: S3 File — (DOCX) [file pone.0334395.s003.docx]

| Supporting Information 3 (S3). Summary of the Mann – Whitney analysis carried out for the Evaluation of the integrity of actin filaments and nuclei by immunofluorescence with the addition of FB1. | | | | | | | | | | |
| --- | --- | --- | --- | --- | --- | --- | --- | --- | --- | --- |
| **Variable**  ANNE  X 2. Summary of the Mann–Whitney analysis for the evaluation of cell morphology in the treatment with DON | **Comparison** | **N1/N2** | **Median 1** | **Median 2** | **ETA1–ETA2** | | **95.5% CI** | **W** | **p-value** | **Adjusted p-value** |
| Loss of nuclear integrity | C vs B | 6 | 0.0 | 1.0 | | -1.0 | (-2.0001; -0.9996) | 23.0 | 0.0131 | 0.0073 |
| Loss of nuclear integrity | C vs M | 6 | 0.0 | 1.5 | | -1.0 | (-3.000; -1.000) | 22.5 | 0.0104 | 0.0067 |
| Loss of nuclear integrity | C vs A | 6 | 0.0 | 3.0 | | -3.0 | (-4.000; -1.000) | 21.5 | 0.0065 | 0.0045 |
| Loss of nuclear integrity | B vs M | 6 | 1.0 | 1.5 | | 0.0 | (-2.000; 1.000) | 34.0 | 0.4712 | 0.4166 |
| Loss of nuclear integrity | B vs A | 6 | 1.0 | 3.0 | | -2.0 | (-2.999; -0.000) | 26.0 | 0.0453 | 0.0358 |
| Loss of nuclear integrity | M vs A | 6 | 1.5 | 3.0 | | -1.0 | (-2.999; 0.000) | 30.0 | 0.1735 | 0.1566 |
| Actin depolymerization | C vs B | 6 | 0.0 | 0.0 | | 0.0 | (-1.0001; 0.0004) | 36.0 | 0.6889 | 0.5948 |
| Actin depolymerization | C vs M | 6 | 0.0 | 0.0 | | 0.0 | (-1.0001; 0.0004) | 36.0 | 0.6889 | 0.5948 |
| Actin depolymerization | C vs A | 6 | 0.0 | 0.5 | | 0.0 | (-1.000; 0.000) | 33.0 | 0.3785 | 0.2824 |
| Actin depolymerization | B vs M | 6 | 0.0 | 0.0 | | -0.0 | (-1.000; 1.000) | 39.0 | 1.0000 | 1.0000 |
| Actin depolymerization | M vs A | 6 | 0.0 | 0.5 | | 0.0 | (-1.000; 1.000) | 36.0 | 0.6889 | 0.6404 |
| Actin depolymerization | B vs A | 6 | 0.0 | 0.5 | | 0.0 | (-1.000; 1.000) | 36.0 | 0.6889 | 0.6404 |
| Loss of cell confluence | B vs M | 6 | 2.0 | 2.0 | | 0.0 | (-1.000; -0.000) | 34.0 | 0.4712 | 0.3865 |
| Loss of cell confluence | B vs A | 6 | 2.0 | 3.5 | | -2.0 | (-3.000; -1.000) | 23.0 | 0.0131 | 0.0092 |
| Loss of cell confluence | M vs A | 6 | 2.0 | 3.5 | | -1.0 | (-2.001; 0.000) | 25.0 | 0.0306 | 0.0228 |
| Lethal cytomorphological change | C vs B | 6 | 0.0 | 1.5 | | -1.0 | (-2.000; -1.000) | 22.5 | 0.0104 | 0.0063 |
| Lethal cytomorphological change | C vs M | 6 | 0.0 | 2.0 | | -2.0 | (-2.0002; -0.9996) | 22.0 | 0.0082 | 0.0049 |
| Lethal cytomorphological change | C vs A | 6 | 0.0 | 3.5 | | -3.0 | (-4.000; -3.000) | 21.0 | 0.0051 | 0.0032 |
| Lethal cytomorphological change | B vs M | 6 | 1.5 | 2.0 | | 0.0 | (-1.000; 1.000) | 36.0 | 0.6889 | 0.6404 |
| Lethal cytomorphological change | B vs A | 6 | 1.5 | 3.5 | | -2.0 | (-3.000; -1.000) | 21.0 | 0.0051 | 0.0039 |
| Lethal cytomorphological change | M vs A | 6 | 2.0 | 3.5 | | -2.0 | (-3.000; -1.000) | 21.0 | 0.0051 | 0.0037 |
| Spindle-shaped characteristic | C vs B | 6 | 4.0 | 3.0 | | 1.0 | (0.000; 2.000) | 53.0 | 0.0306 | 0.0183 |
| Spindle-shaped characteristic | C vs M | 6 | 4.0 | 2.0 | | 2.0 | (1.000; 3.000) | 57.0 | 0.0051 | 0.0035 |
| Spindle-shaped characteristic | C vs A | 6 | 4.0 | 1.0 | | 3.0 | (2.000; 4.000) | 57.0 | 0.0051 | 0.0035 |
| Spindle-shaped characteristic | B vs M | 6 | 3.0 | 2.0 | | 1.0 | (0.000; 2.000) | 53.0 | 0.0306 | 0.0183 |
| Spindle-shaped characteristic | B vs A | 6 | 3.0 | 1.0 | | 2.0 | (1.000; 3.000) | 57.0 | 0.0051 | 0.0035 |
| Spindle-shaped characteristic | M vs A | 6 | 2.0 | 1.0 | | 1.0 | (0.000; 2.000) | 53.0 | 0.0306 | 0.0183 |
| Spindle-shaped characteristic | C vs A | 6 | 1.0 | 3.5 | | -3.0 | (-3.000; -2.000) | 21.0 | 0.0051 | 0.0034 |

Comparison column: A- FB1-HD at 48 h; M-FB1-MD at 48 h; B-FB1-LD at 48 hours; C- control treatment without addition of mycotoxin; vs- versus or comparison.
