# Supplementary material for: Generation of a primary culture of chick embryo enterocytes to evaluate the effects of fumonisin B1 and deoxynivalenol on cell morphology, actin filaments and nuclei
Source: PLoS One. 2025 Dec 11;20(12):e0334395. doi: 10.1371/journal.pone.0334395 (PMC12697969; doi:10.1371/journal.pone.0334395)
Supplement: S4 File — (DOCX) [file pone.0334395.s004.docx]

| Supporting Information 4 (S4). Summary of the Mann–Whitney analysis for the evaluation of actin filament and nuclear integrity by immunofluorescence following DON exposure. | | | | | | | | | |
| --- | --- | --- | --- | --- | --- | --- | --- | --- | --- |
| **Variable evaluated** | **Comparison** | **N1/N2** | **Median 1** | **Median 2** | **ETA1–ETA2** | **95.5% CI** | **W** | **p-value** | **Adjusted p-value** |
| Loss of cell confluence | C vs B | 6 | 0.0 | 2.0 | -2.0 | (-3.000; -1.000) | 21.0 | 0.0051 | 0.0035 |
| Loss of cell confluence | C vs M | 6 | 0.0 | 3.0 | -3.0 | (-4.000; -2.000) | 21.0 | 0.0051 | 0.0035 |
| Loss of cell confluence | C vs A | 6 | 0.0 | 4.0 | -4.0 | (-4.0002; -2.9996) | 21.0 | 0.0051 | 0.0030 |
| Loss of cell confluence | B vs M | 6 | 2.0 | 3.0 | -1.0 | (-2.000; -0.000) | 25.0 | 0.0306 | 0.0183 |
| Loss of cell confluence | B vs A | 6 | 2.0 | 4.0 | -2.0 | (-2.0002; -0.9996) | 22.0 | 0.0082 | 0.0049 |
| Loss of cell confluence | M vs A | 6 | 3.0 | 4.0 | -1.0 | (-1.0002; 0.0004) | 30.0 | 0.1735 | 0.1122 |
| Actin filament polymerization | actinCa vs B | 6 | 0.0 | 0.5 | 0.0 | (-1.000; 1.000) | 36.0 | 0.6889 | 0.6404 |
| Actin filament polymerization | actinCa vs M | 6 | 0.0 | 0.0 | -0.0 | (-1.000; 1.000) | 39.0 | 1.0000 | 1.0000 |
| Actin filament polymerization | actinCa vs A | 6 | 0.0 | 0.0 | -0.0 | (-1.000; 1.000) | 39.0 | 1.0000 | 1.0000 |
| Actin filament polymerization | actinBa vs M | 6 | 0.5 | 0.0 | 0.0 | (-1.000; 1.000) | 42.0 | 0.6889 | 0.6404 |
| Actin filament polymerization | actinBa vs A | 6 | 0.5 | 0.0 | 0.0 | (-1.000; 1.000) | 42.0 | 0.6889 | 0.6404 |
| Actin filament polymerization | actinMa vs A | 6 | 0.0 | 0.0 | -0.0 | (-1.000; 1.000) | 39.0 | 1.0000 | 1.0000 |
| Loss of nuclear integrity | C vs B | 6 | 0.0 | 2.5 | -2.0 | (-3.000; -1.000) | 21.0 | 0.0051 | 0.0037 |
| Loss of nuclear integrity | C vs M | 6 | 0.0 | 3.0 | -3.0 | (-4.000; -2.000) | 21.0 | 0.0051 | 0.0035 |
| Loss of nuclear integrity | C vs A | 6 | 0.0 | 4.0 | -4.0 | (-4.0002; -2.9996) | 21.0 | 0.0051 | 0.0030 |
| Loss of nuclear integrity | B vs M | 6 | 2.5 | 3.0 | -1.0 | (-2.000; -0.000) | 27.0 | 0.0656 | 0.0379 |
| Loss of nuclear integrity | B vs A | 6 | 2.5 | 4.0 | -1.0 | (-2.000; -1.000) | 22.5 | 0.0104 | 0.0063 |
| Loss of nuclear integrity | M vs A | 6 | 3.0 | 4.0 | -1.0 | (-1.0002; 0.0004) | 30.0 | 0.1735 | 0.1122 |
| Spindle-shaped characteristic | C vs B | 7 | 4.0 | 3.5 | 0.0 | (-0.0002; 0.9998) | 58.5 | 0.4822 | 0.4236 |
| Spindle-shaped characteristic | C vs M | 7 | 4.0 | 2.0 | 2.0 | (1.0001; 1.9999) | 75.0 | 0.0049 | 0.0029 |
| Spindle-shaped characteristic | C vs A | 7 | 4.0 | 2.0 | 2.0 | (1.0001; 1.9999) | 75.0 | 0.0049 | 0.0029 |
| Spindle-shaped characteristic | B vs M | 7 | 3.5 | 2.0 | 1.0 | (0.9998; 1.9998) | 74.0 | 0.0073 | 0.0048 |
| Spindle-shaped characteristic | B vs A | 7 | 3.5 | 2.0 | 1.0 | (0.9998; 1.9998) | 74.0 | 0.0073 | 0.0048 |
| Spindle-shaped characteristic | M vs A | 7 | 2.0 | 2.0 | -0.0 | (-1.0001; 1.0001) | 52.5 | 1.0000 | 1.0000 |
| Lethal cytomorphological change | C vs B | 7 | 0.0 | 2.0 | -2.0 | (-2.4996; -0.9998) | 28.0 | 0.0022 | 0.0015 |
| Lethal cytomorphological change | C vs M | 7 | 0.0 | 3.0 | -3.0 | (-3.500; -2.000) | 28.0 | 0.0022 | 0.0017 |
| Lethal cytomorphological change | C vs A | 7 | 0.0 | 4.0 | -3.5 | (-4.0000; -3.0001) | 28.0 | 0.0022 | 0.0012 |
| Lethal cytomorphological change | B vs M | 7 | 2.0 | 3.0 | -1.0 | (-1.500; -0.000) | 36.5 | 0.0476 | 0.0313 |
| Lethal cytomorphological change | B vs A | 7 | 2.0 | 4.0 | -2.0 | (-2.0000; -0.9998) | 29.0 | 0.0033 | 0.0016 |
| Lethal cytomorphological change | M vs A | 7 | 3.0 | 4.0 | -1.0 | (-1.000; -0.000) | 35.0 | 0.0298 | 0.0173 |

Comparison column: A- FB1-HD at 48 h; M-FB1-MD at 48 h; B-FB1-LD at 48 hours; C- control treatment without addition of mycotoxin; vs- versus or comparison.
